# Mesenchymal stem cell-derived extracellular vesicles: current advances in preparation and therapeutic applications for neurological disorders

**DOI:** 10.3389/fcell.2025.1626996

**Published:** 2025-08-18

**Authors:** Shuang Li, Jiayi Zhang, Luyao Sun, Ze Yang, Xinxing Liu, Jianling Liu, Xifu Liu

**Affiliations:** ^1^ Ministry of Education Key Laboratory of Molecular and Cellular Biology, Hebei Anti-Tumour Molecular Target Technology Innovation Center, College of Life Science, Hebei Normal University, Shijiazhuang, China; ^2^ Jianyuan Precision Medicines (Zhangjiakou) Co., Ltd., Zhangjiakou, China; ^3^ Shijiazhuang Zhuohan Biological Technology Co., Ltd., Shijiazhuang, China; ^4^ Guangxi Academy of Sciences, Nanning, China

**Keywords:** mesenchymal stem cell-derived extracellular vesicles (MSC-EVs), preparation, characterization, pharmacokinetics, neurological disorders

## Abstract

Extracellular vesicles (EVs), nanoscale vesicles released by various cell types, have garnered significant attention in regenerative medicine. Mesenchymal stem cell-derived EVs (MSC-EVs) exhibit unique advantages, including their compact size, ability to traverse the blood-brain barrier (BBB), low immunogenicity, and high biosafety profile. However, challenges such as standardization of isolation protocols, establishment of quality control criteria, and scalability of production remain unresolved. This review critically examines the methodologies for preparation, characterization, and pharmacokinetic profiling of MSC-EVs, alongside their therapeutic potential in neurological disorders. By synthesizing current advancements, this work aims to elucidate the translational value of EVs in clinical practice. Additionally, it seeks to accelerate their transition from preclinical research to therapeutic applications, and provide a robust theoretical foundation for novel strategies in treating neurological diseases.

## 1 Introduction

Extracellular vesicles (EVs), nanoscale membrane-bound particles released by diverse cell types including fibroblasts, immune cells (e.g., T cells, B cells, dendritic cells), adipocytes, stem cells, and tumor cells, have emerged as critical mediators of intercellular communication. These vesicles are ubiquitously present in all bodily fluids, such as blood, urine, breast milk, amniotic fluid, and bronchoalveolar lavage fluid (Kimiz-Gebologlu and Oncel, 2022). EVs hold immense potential in clinical diagnostics and therapeutics, with mesenchymal stem cell-derived EVs (MSC-EVs) representing a novel paradigm for disease intervention.

Stem cells encompass multiple subtypes, notably mesenchymal stem cells (MSCs), induced pluripotent stem cells (iPSCs), hematopoietic stem cells (HSCs), and others. Unlike other stem cell types, MSCs exhibit broader tissue distribution, superior multipotency, and enhanced self-renewal capacity (Li J. et al., 2023). MSCs secrete heterogeneous EV populations, primarily classified into EVs (30–150 nm in diameter), microvesicles (150–500 nm in diameter), and apoptotic bodies (500–800 nm in diameter) based on biogenesis pathways and size (Patel et al., 2024). MSC-EVs are regarded as important paracrine mediators for information transfer between MSCs and their target cells. Initially, MSC-EVs were underappreciated due to limited understanding of their biological relevance (Guo et al., 2020; Johnstone et al., 1987). However, accumulating research has demonstrated that MSC-EVs possess distinct functional properties. For instance, they can accurately reflect the specific state of their parental cells (Rhim et al., 2023). They also play roles in maintaining cellular homeostasis, anti-inflammation, tissue repair, immunomodulation, and other aspects (Drobiova et al., 2023). Additionaslly, MSC-EVs can efficiently deliver exogenous chemicals and biomolecules, serving as a novel and promising drug delivery platform and opening up new avenues for regenerative medicine (Miron et al., 2024).

**FIGURE 1:**
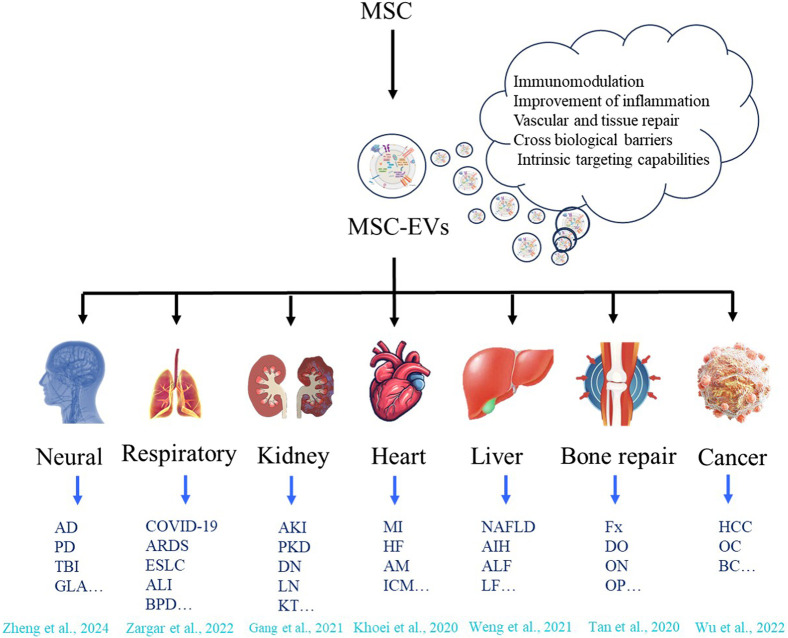
Therapeutic applications of MSC-EVs across diverse disease types. MSC can release MSC - EVs with capabilities of immunomodulation, improvement of inflammation, vascular and tissue repair, crossing biological barriers, and intrinsic targeting (Hade et al., 2021; Zheng et al., 2024; Zargar et al., 2022; Gang et al., 2021; Khoei et al., 2020; Weng et al., 2021; Tan et al., 2020; Wu et al., 2022). These vesicles show therapeutic application potential in various disease types, including neurological diseases (such as Alzheimer’s disease (AD), Parkinson’s disease (PD), Traumatic brain injury (TBI), Glaucoma (GLA) etc.); respiratory diseases (such as COVID-19, Acute respiratory distress syndrome (ARDS), Early-staged lung cancer (ESLC), Acute lung injury (ALI), Broncho-pulmonary dysplasia (VPD) etc.); kidney diseases (such as Acute kidney injury (AKI), Polycystic kidney disease (PKD), Diabetic nephropathy (DN), Lupus nephritis (LN), Kidney transplant (KT) etc.); heart diseases (such as Myocardial infarction (MI), Heart failure (HF), Acute myocarditis (AM), Ischemic cardiomyopathy (ICM) etc.); liver diseases (such as non-alcoholic fatty liver disease (NAFLD), autoimmune hepatitis (AIH), Acute liver failure (ALF), Liver Fibrosis (LF) etc.); bone repair (such as Fracture (Fx), Distraction osteogenesis (DO), Osteonecrosis (ON), Osteoporosis (OP) etc.), and cancers (such as Hepatocellular carcinoma (HCC), Ovarian cancer (OC), Ovarian cancer (OC), Breast cancer (BC) etc.).

Compared with EVs from other sources, EVs derived from MSCs have several potential therapeutic advantages. This is mainly due to a series of novel and beneficial properties (Table 1). For example, they are smaller in size, enabling a more extensive and precise distribution *in vivo*. They have low immunogenicity, which makes them less likely to cause rejection reactions and reduces potential risks. They lack a cell nucleus, effectively preventing the risk of tumor transformation. They have higher stability and can maintain their activity under different environmental conditions (Chattopadhyay et al., 2025). They are easier to produce, improving the feasibility of their clinical application. They can be preserved for a long time, providing convenience for the storage and transportation of drugs. Moreover, they also have great potential to load proteins, small molecules, or RNAs to deliver biomolecules (Xie et al., 2023; Lotfy et al., 2023; Muskan et al., 2024). In addition to other bioactive molecules, more than 304 proteins and 150 microRNAs have been found in MSC-EVs (Lotfy et al., 2023). All these bioactive molecules have a good therapeutic effect on tissue recovery by maintaining and recruiting endogenous stem cells, inhibiting apoptosis, regulating the immune system, and stimulating angiogenesis (Hassanzadeh et al., 2021). Over the past decades, preclinical and clinical studies have validated the therapeutic efficacy of MSC-EVs across diverse pathologies, including neurological disorders, respiratory diseases, kidney diseases, heart diseases, liver diseases, bone defects, and malignancies (Figure 1). Here, we focus on the applications of MSC-EVs derived from different sources in the content of neurological disorders.

**TABLE 1:** Unique properties of MSC-EVs and potential therapeutic applications.

MSC-EVs Property	Potential therapeutic benefits	Reference
Smaller particle size	Enable broader and more precise targeted distribution	Shi and Esfandiari (2022)
Low immunogenicity	Reduces the probability of immune rejection, thereby minimizing associated potential risks	Li et al. (2022)
Lack of nuclear structure	Effectively avoid the potential risk of tumorigenic transformation	Dabrowska et al. (2021)
High biological stability	Maintain biological activity under diverse environmental conditions	Chattopadhyay et al. (2025)
Simpler preparation process (vs. MSCs)	It is easy for large-scale production, which enhances its practical feasibility in clinical application scenarios	Johnson et al. (2021)
Capacity for long-term storage	Provides convenient conditions for drug storage and transportation	Gholami et al. (2021)
Enrichment with bioactive molecules	Exhibits excellent efficacy in tissue repair and regeneration	Zheng et al. (2024)

## 2 Preparation of MSC-EVs

MSC-EVs exhibit heterogeneous diameters ranging from tens to hundreds of nanometers, with their size distribution being method-dependent during isolation (Patel et al., 2019). This section summarizes common preparation techniques, compares their merits and limitations, and provides guidance for selecting optimal protocols under specific experimental conditions.

### 2.1 Centrifugation-based methods

#### 2.1.1 Differential ultracentrifugation

Differential ultracentrifugation remains a gold standard for EVs isolation. The protocol involves sequential centrifugation steps with increasing centrifugal forces to remove cellular debris and subcellular components, followed by ultracentrifugation (≥100,000 × g) to pellet EVs. Initial low-speed centrifugation eliminates intact cells and large debris, while subsequent high-speed steps clear smaller contaminants. Final ultracentrifugation precipitates EVs, with optional repeat cycles to enhance purity (Aliakbari et al., 2024; Tiszbein et al., 2025).

The EVs isolated by the differential ultracentrifugation method have a relatively high purity. This method requires expensive specialized equipment such as an ultracentrifuge, with the centrifugal speed needing to reach 100,000 × g. EVs can be damaged due to multiple centrifugations at excessively high speeds. During the extraction process, experimenters may perform multiple ultracentrifugations, which may cause certain damage to the structure of EVs.

#### 2.1.2 Density gradient centrifugation

Density gradient centrifugation is an improved method of traditional differential ultracentrifugation. EVs are separated based on buoyant density differences using continuous or discontinuous gradients of sucrose, iodixanol, or cesium chloride. Particles migrate to equilibrium positions matching their densities during centrifugation.

The density gradient centrifugation method is an important separation technique. The number of ultracentrifugations can be reduced, thereby maintaining the integrity of vesicles, enabling the separation of different types of extracellular vesicles, and it has been widely applied in fields such as biology and medicine (Auquière et al., 2025; Chen et al., 2019; D'Acunzo et al., 2022). This method can improve the purity of the separated EVs (Langevin et al., 2019). However, this method has a complex operation process, further washing is required to remove the density gradient medium from the EVs suspension, resulting in a low yield of EVs. It requires a high level of technical skills from the operators, is time-consuming, and cannot effectively remove lipoproteins and chylomicrons from blood samples (Wang C. C. et al., 2024).

### 2.2 Ultrafiltration

Ultrafiltration employs size-exclusion membranes under pressure gradients to concentrate EVs. Variants include sequential filtration, centrifugal ultrafiltration, and tangential flow filtration (Lai et al., 2022; Busatto et al., 2018; Xu et al., 2015). These methods use membrane filters with specific molecular weight or size exclusion limits to separate suspended particles or polymers according to their size or molecular weight.

Ultrafiltration achieves separation mainly through the pore size sieving effect of the membrane, eliminating the need for introducing chemical substances such as precipitants, organic solvents, and chelating agents, thus reducing chemical contamination and its impact on the contents of EVs (Yang et al., 2020). The disadvantage is that membrane adhesion reduces the yield of EVs. The pressure and shear force during filtration may damage the morphology of EVs. Under the premise of reasonably controlling the pressure, the ultrafiltration method can minimize damage to the EVs’ morphology. Additionally, the filter membrane is prone to damage or blockage, which affects the separation efficiency.

### 2.3 Anion exchange chromatography (AEC)

AEC is a reliable method for preparing EVs, which utilizes the interaction between the inherent negative charges of EVs and the charged packing materials inside the chromatographic column. This technique involves several key steps: preparation of reagents, materials, and instrumental equipment; equilibration of the chromatographic column; sample loading, elution of EVs, and collection (Wang S. et al., 2024). Since this method can concentrate samples, it is easy to be combined with other purification methods (such as ultrafiltration and tangential flow filtration) to construct a two-dimensional purification strategy based on differences in surface charge and size (Saari et al., 2023). It was found that the combination of AEC with size exclusion chromatography (SEC) can improve the sample purity of EVs compared with the use of SEC alone (Wang H. et al., 2023). Seo et al. concentrated and deproteinized the culture supernatants by ultrafiltration, then performed AEC, which enabled the acquisition of a large quantity of high-purity EVs without reducing their biological activity (Seo et al., 2022). Pirolli et al. successfully combined tangential flow filtration with AEC to purify EVs, thus improving the purity (Pirolli et al., 2023).

### 2.4 Size exclusion chromatography (SEC)

SEC is a well-established method for separating macromolecules based on their molecular size or hydrodynamic volume. A standard SEC configuration consists of a porous stationary phase for chromatographic separation, which can optionally be coupled with a pump for elution. According to the size and shape of EVs, separation is achieved using the porous packing material in the chromatographic column. Molecules of different sizes have different retention times in the column, and EVs are eluted within a specific time frame (Al-Madhagi, 2024). SEC has been widely used to separate EVs from different sample matrices of prokaryotes and eukaryotes, including specimens derived from cell cultures, blood samples (Liangsupree et al., 2021), urine (Gan et al., 2022), saliva (Han et al., 2023), and tears (Aqrawi et al., 2019).

SEC has several advantages, such as simple operation, good reproducibility, the ability to handle large-volume samples, and the relatively uniform size of the separated EVs. However, its drawback is that it may separate particles with similar sizes to other particles, leading to a decrease in purity. Currently, commercial chromatographic columns for separating and purifying EVs have been developed based on the SEC principle. For example, the qEV chromatographic column, a commercial product from iZON based on SEC, can extract high concentration EVs from an initial sample volume ranging from 150 μL to 10 mL within 15 min. It ensures the stability of the biochemical components and morphological structure of EVs while achieving efficient extraction (Reseco et al., 2024).

### 2.5 Polymer precipitation

Polymer-based coprecipitation is a commonly used strategy in commercial EVs separation kits, such as ExoQuick™ (System Biosciences, United States), ExoPrep (HansaBioMed, Estonia), and Total Exosome Isolation™ (Invitrogen, United States). Among various hydrophilic polymers, Polyethylene glycol (PEG) is widely used as a precipitation reagent. Hydrophilic PEG can interact with the water molecules surrounding EVs, thus forming a hydrophobic microenvironment. During this process, the solubility of EVs decreases, and they will precipitate under low-speed centrifugation (Staubach et al., 2021). These commercial kits have a high yield and are easy to adapt to different studies. At the same time, this method avoids expensive ultracentrifugation and reduces damage to EVs. However, it is worth noting that the polymer precipitation process may coprecipitate proteins, nucleic acids, and lipids (Xu et al., 2022).

Due to the simplicity of this method, commercially available kits for separating EVs by PEG precipitation have also been developed. The processing time of PEG precipitation is relatively short, but it faces challenges such as low purity and recovery rate, and it is difficult to remove PEG in the last step. Therefore, this method is rarely used alone nowadays and is usually an auxiliary method, and is not recommended for clinical use due to the presence of non-EVs proteins, immunoglobulins, viral particles, immune complexes, and other contaminants in addition to EVs in the final pellet obtained from the PEG-based EVs isolation process (Welsh et al., 2024; Sidhom et al., 2020).

### 2.6 Immunomagnetic beads

This method utilizes specific markers on the surface of EVs. By interacting with magnetic beads conjugated with specific antibodies, and under the action of an external magnetic field, the magnetic beads carrying EVs are separated from other components, thus achieving the separation and purification of EVs. Magnetic particles such as iron, nickel, neodymium, or magnetite can be easily functionalized with biomolecules (such as antibodies). This modification enables the magnetic particles to specifically attach to EVs, facilitating the extraction of EVs from complex matrices through magnetic drive. This method helps to eliminate the interference caused by the biological fluid matrix, achieve the preconcentration of EVs, and improve the sensitivity of the detection process (Lima Moura et al., 2020).

The immunomagnetic bead method is a commonly used approach for preparing EVs and has the following advantages. High specificity: By selecting antibodies against specific markers of EVs, highly specific separation of EVs can be achieved. For instance, antibodies agianst CD9, CD 63 and CD81 can be used for purification of EVs (Cheng et al., 2025; Allelein et al., 2022; Li D. et al., 2024). High purity: Through multiple steps of washing and magnetic separation, most of the impurities can be removed, obtaining EVs with a relatively high purity. Relatively simple operation: The operation steps of the immunomagnetic bead method are relatively simple, and it does not require complex equipment and techniques. However, overall, it has a high cost: The cost of antibodies and magnetic beads is relatively high, increasing the experimental expenses. There may be non-specific binding: In some cases, the magnetic beads may have non-specific binding with other components in the sample, affecting the purity of EVs. High requirements for antibody selection: EVs from different sources may have different marker expressions, and appropriate antibodies or a combination of several antibodies need to be selected to effectively separate EVs (Lane et al., 2017; Peng et al., 2025; Liu C. et al., 2024; Zhang et al., 2025; He et al., 2017).

### 2.7 Microfluidic technologies

Traditional methods for the separation and purification of EVs often require a large amount of samples, and the operation is complex and time-consuming. Microfluidic technology, with its unique advantages such as adjustability, diverse material options, low cost, high processing efficiency, and minimal sample requirement, provides an ideal platform for precision medicine applications (Lan et al., 2025). Microfluidics is an EVs extraction technology based on signal detection, which specifically targets major protein markers on EVs for EVs purification. This innovative method can effectively separate and allow visualization of EVs enriched on the surface of microspheres, and is capable of manipulating tiny fluids in microtubes (with volumes ranging from a few microliters to several hundred microliters) (Wang W. et al., 2024; Theel and Schwaminger, 2022). Woo et al. developed a procedure consisting of two filtration chambers (with pore sizes of 600 nm and 20 nm respectively) for the automatic enrichment and separation of EVs and other vesicles from biological samples, which requires only low centrifugal force (<500 g) and can complete the enrichment process quickly (Woo et al., 2017). The microfluidic chip developed by Hassanpour Tamrin et al. has unique advantages such as fast separation speed, high throughput, and low sample requirement, making it very suitable for separating EVs from small amounts of precious biological sample (Hassanpour Tamrin et al., 2021). Suwatthanarak et al. modified antibodies, aptamers, or peptides in microfluidic channels to improve the specificity of EVs separation (Suwatthanarak et al., 2021). Yu et al. reported a highly integrated EVs separation and detection chip (EVs SD). This chip, modified with anti-CD63 antibodies, can effectively separate EVs from the cell culture supernatants and clinical sera of gastric cancer patients (stages I and II) (Yu et al., 2021). Despite the continuous development and expanding application scope of microfluidic separation technologies, their popularity in standardized laboratories remains relatively limited, and the application of these technologies requires specific professional knowledge and dedicated preparation equipment as support (Wu et al., 2023).

### 2.8 Ultrafast-isolation system: EXODUS

The EXODUS employs a dual-frequency harmonic oscillator integrated with negative pressure oscillation (NPO) to isolate EVs from biofluids (e.g., urine, plasma, tears) (Chen et al., 2021). This system utilizes nanoporous membranes subjected to high-frequency (5–8 kHz) piezoelectric vibration and low-frequency (∼200 Hz) mechanical oscillation, coupled with alternating pressure gradients. The synergistic effect minimizes membrane fouling while enhancing particle resuspension, enabling rapid, clog-free processing of samples (Chen et al., 2021; Hu et al., 2022). Key advantages include exceptional EVs recovery (>90%), high purity (>98% contaminant removal). Additionally, different models of EXODUS can cover processing volumes ranging from 10 µL to 10 L, while still maintaining the integrity of EVs, enabling downstream molecular analysis (Hu et al., 2023; Jiang S. et al., 2024).

EXODUS facilitates high-yield EV isolation for biomarker discovery across multiple disease contexts. It enables non-invasive detection of Alzheimer’s-associated proteins (Aβ, tau) in blood EVs at zeptomolar sensitivity (Zheng et al., 2025), stratifies prostate cancer risk via urinary EV RNA signatures (EPS model: FOXA1/PCA3/KLK3), reducing unnecessary biopsies by 26% (Jiang W. et al., 2024), and identifies ocular disorder biomarkers in tear EVs (Hu et al., 2022). However, its adoption faces limitations, including dependency on specialized equipment, variable performance with viscous samples requiring pre-filtration (Chen et al., 2021). Cost constraints of disposable cartridges further challenge large-scale implementation (Hu et al., 2023).

In summary, EVs isolation initially relied on ultracentrifugation, which remains the current gold standard. To address its limitations, alternative techniques have been developed, based on EVs biophysical and biochemical properties such as size or surface markers (Jia et al., 2022). However, these methods still have issues with specific isolation, yield, and purity. This is partly due to EVs heterogeneity and the co-isolation of non-EV contaminants like lipoproteins and uromodulin (Doyle and Wang, 2019; Théry et al., 2018) (Table 2). Method selection depends on research or clinical needs. For example, SEC, despite being prone to serum protein contamination, offers high yield and is suitable for large-sample studies (An et al., 2018). Isolation techniques also affect EVs function: SEC-isolated EVs, for instance, show a stronger effect in promoting endothelial migration than those isolated by ultracentrifugation, though this difference disappears when normalized by particle number (Takov et al., 2018). Thus, choosing optimal isolation methods, combined with multi-dimensional detection, is critical for the clinical translation of EVs. While combining multiple methods can improve purity and yield, it increases costs and complexity, which hinders clinical application. Developing rapid, efficient, and reproducible techniques remains a key challenge (Kimiz-Gebologlu and Oncel, 2022).

**TABLE 2:** Comparative analysis of EVs isolation methods.

Method	Advantages	Limitations
Differential Ultracentrifugation	It is the gold standard for extracting EVs, with relatively high purity, and is a commonly used basic method in laboratories	It requires expensive equipment such as an ultracentrifuge, and multiple centrifugations may cause damage to or loss of the EVs structure, so the number of centrifugations needs to be considered
Density Gradient Centrifugation	High resolution and improved purity	The operation is complex, and further washing will lead to a low yield of EVs. It has high technical requirements for operators, time-consuming, and there is a problem of relatively low purity (blood samples)
Ultrafiltration	The steps are relatively simple, the time required is short, and the impact on the contents of EVs is minimal	Membrane adhesion reduces the yield, and pressure and shear forces may cause morphological changes
AEC	High purity	Used in combination with other methods
SEC	Simple operation, good reproducibility, and capable of handling a large number of samples	Low purity
Polymer Precipitation	Reduces damage and is time-saving	Low purity and low recovery rate
Immunomagnetic Beads	The operation is relatively simple, and the purity is high	High cost and there is non-specific binding
Microfluidics	Fast separation speed, high throughput, and requires less sample	Requires specific professional knowledge and dedicated preparation equipment
EXODUS	It has advantages in the isolation efficiency of EVs, and its automated operation process significantly improves the repeatability of extraction results	The reliance on specialized equipment will increase the economic cost of the experiment

## 3 Characterization of EVs

Obtaining high-quality EVs poses certain challenges. The identification of EVs is critical for EVs-related research and applications. This step is mainly aimed at determining whether the extracted particles are EVs, so downstream analysis becomes particularly crucial. To date, the main downstream analysis methods include electron microscopy imaging, Nanoparticle Tracer Analysis (NTA), Western blotting (WB), Enzyme-Linked Immunosorbent Assay (ELISA), Flow Cytometry (FCM), and proteomics techniques (Ströhle et al., 2022; Lai et al., 2024; Welsh et al., 2024). By applying these analytical techniques, the molecular components of EVs can be discovered, which provides a basis for the management of EVs (Szatanek et al., 2015).

### 3.1 Electron microscopy

Techniques such as Transmission Electron Microscopy (TEM) and Scanning Electron Microscope (SEM) in electron microscopy can perform high-resolution imaging of the morphology and size distribution of EVs. TEM is a widely used technique and is crucial for evaluating the quality and purity of samples containing EVs. This technique can distinguish between individual EVs and particles of a similar size to EVs (Klaihmon et al., 2023). TEM can provide a detailed view of the ultrastructure of EVs, revealing their lipid bilayer membranes and internal cargo. SEM offers 3D imaging capabilities, which helps in visualizing the surface morphology of EVs (Ju et al., 2022). Electron microscopy is invaluable for confirming the identity of EVs and assessing their structural integrity (Colombo et al., 2021).

### 3.2 NTA

NTA is another key technology used for quantifying and determining the size of EVs. This technique demonstrates good reproducibility and accuracy when measuring the particle concentration and determining the particle size distribution of EVs isolated from different sources through various methods (Comfort et al., 2021). NTA employs laser scattering to monitor the movement of EVs, which helps in the immediate examination of their size changes and diversity. NTA is a rapid and sensitive method for quantifying EVs, but it may be affected by factors such as particle aggregation and sample viscosity (Colombo et al., 2021). Therefore, in order to further improve the reproducibility of EVs research, the standardization of the NTA analysis method is essential.

### 3.3 WB

WB is a technique used to identify the presence of protein markers on EVs. It requires the analyte to have a relatively high purity and concentration, and it can quantitatively and qualitatively analyze specific proteins (Torres et al., 2024). Researchers typically use transmembrane protein families (CD9, CD63, CD81), heterotrimeric G protein subunit β-like proteins (Alix), tumor susceptibility gene 101 (TSG101), and syntenin-1, etc. as positive markers; endoplasmic reticulum chaperone protein Calnexin, Golgi matrix protein GM130, etc. as negative markers to identify the presence of EVs (Li P. et al., 2024; Khanabdali et al., 2024).

### 3.4 ELISA

ELISA has high detection sensitivity, convenient operation, and a fast detection speed, and it can be scaled up for higher-throughput measurements (Karachaliou et al., 2023). ELISA is a well-known quantitative analysis technique. It achieves colorimetric changes to indicate antigen-antibody interactions by incorporating enzyme-linked conjugates and enzyme substrates (Islam et al., 2023). Therefore, this method requires a pair of non-interacting antibodies, and it has higher requirements for detection specificity. It can only analyze EVs at the individual level. The determination can quantify EVs derived from specific cells based on the selection of the primary antibody, and this detection method is usually single.

### 3.5 FCM

FCM is an instrument used to measure the fluorescence and light scattering signals of individual particles flowing through a focused light source (most commonly a laser beam) in a liquid suspension (Welsh et al., 2023), capable of detecting and characterizing individual particles at a flux of thousands of particles per second. FCM allows for the quantitative assessment of EVs by detecting their size, surface markers, and fluorescence content. Fluorescent dyes or antibodies targeting specific surface antigens label the EVs, which are then analyzed using a flow cytometer equipped with appropriate detectors (Bettin et al., 2023). FCM is capable of performing multiparametric analysis of EVs subpopulations and is compatible with high-throughput screening applications (Kuiper et al., 2021).

### 3.6 Proteomic profiling

Compared with the small-scale analysis carried out by WB and ELISA, proteomics technology has obvious advantages. The term “proteomics” is used to describe the study of a large number of proteins in biological complexes, including cells, tissues, organelles, vesicles, or protein complexes (Abyadeh et al., 2024). Through proteomics determination, the protein composition of EVs can be comprehensively reflected. In the era of omics science, many researchers have utilized these methods to identify reliable tissue biomarkers. Particularly in the field of proteomics, studies on the protein content or quality of EVs have attracted attention (Aparicio et al., 2024). Through proteomic analysis, the protein composition of EVs can be comprehensively reflected, and their roles under different physiological and pathological conditions can be understood (Abyadeh et al., 2024). It is also possible to explore the differences in various biomolecules encapsulated or carried within EVs, and their associations with differences in sources of MSCs, *in vitro* aging caused by passaging, and differences in in vitro functional assays (Liu Y. et al., 2024). Giuliani et al. demonstrated that proteomic analysis can provide proteomic profiles to study differences in EVs proteins, while enabling the identification of new biomarkers (Giuliani et al., 2024). Martinez-Zalbidea identified 35 proteins through proteomic analysis of EVs and detected a variety of cargo components with potential therapeutic relevance (Martinez-Zalbidea et al., 2025).

#### 3.6.1 Mass spectrometry (MS)

MS is an effective method for detecting and measuring the molecules (such as proteins, lipids, and nucleic acids) carried in EVs. MS-based proteomics, lipidomics, and nucleic acid sequencing provide comprehensive insights into the composition and molecular characteristics of EVs (Burton et al., 2023). Therefore, it enables higher-throughput quantitative analysis of EVs and also allows for the qualitative analysis of the molecular components of EVs. However, it should be noted that MS analysis requires samples to have a relatively high purity, because the salts present in biological fluids and other proteins such as lipoproteins can cause deviations in the analysis results (Pocsfalvi et al., 2016).

#### 3.6.2 Liquid chromatography-MS (LC-MS)

LC-MS has become an effective method for a meticulous and rapid proteomic examination of EVs (Pei et al., 2024). LC separates proteins according to their hydrophobicity or size and is combined with MS detection for identification and quantitative analysis. LC-MS helps to identify a large number of proteins in EVs samples, including low-abundance proteins and proteins with post-translational modifications. This technique is highly suitable for discovery-based proteomics and the quantitative analysis of protein expression changes in AD (Chang et al., 2024).

In practice, multiple methods are required for accurate EVs identification. This is to ensure that the quality of EVs meet the desired requirements (Table 3).

**TABLE 3:** Standardized techniques for EVs characterization.

Characterization technique	Primary application
TEM/SEM	Can perform high-resolution imaging of the morphology and size distribution of EVs
NTA	Measure the particle concentration and particle size distribution
WB	Identify markers to determine purity and concentration
ELISA	Perform quantification through the binding of antigen and antibody
FCM	Quantitatively evaluate EVs by detecting the size, surface markers, and fluorescence content of EVs
MS	Analyze the composition and molecular characteristics
LC-MS	Analyze the composition and molecular characteristics and conduct quantitative analysis

## 4 Pharmacokinetics of EVs

Emerging studies have highlighted the unique therapeutic properties of EVs, including anti-inflammatory, anti-apoptotic, and regenerative capabilities, which offer offering immense therapeutic potential in clinical applications (Jahanbani et al., 2023). The biogenesis of EVs is a highly orchestrated process involving diverse molecular machinery that governs their formation, trafficking, and secretion (Akbari et al., 2020). To elucidate their biological functions and facilitate the development of EV-based therapies, a thorough understanding of EVs pharmacokinetics encompassing biodistribution, cellular uptake, retention, and clearance is imperative (Cheng and Kalluri, 2023).

### 4.1 Administration routes

EVs are naturally distributed through biological fluids including blood, urine, breast milk, cerebrospinal fluid, amniotic fluid, ascites, saliva, bile, and bronchoalveolar lavage fluid enabling targeted delivery to specific cells and tissues (Kowal et al., 2014; Li et al., 2025). Researchers have explored diverse administration routes to optimize therapeutic efficacy across disease models (Table 4).

**TABLE 4:** Comparisition of different administration routes.

Administration route	Distribution characteristics	Half-life	Characteristics	Bioavailability	Reference
Intravenous injection (IV)	Systemic distribution with blood circulation	Relatively short	The most widely used; can cross the blood-brain barrier	Higher	Sandoval and Badiavas (2025), Su et al. (2025), Gupta et al. (2021)
Intranasal administration (IN)	Mainly distributed in the respiratory tract and central nervous system, with low systemic exposure	Longer half-life (lungs)	Easy to operate; more suitable for respiratory diseases	High local bioavailability in the respiratory tract	Su et al. (2025), Rather et al. (2023), Fröhlich (2021), Haney et al. (2020), Sun et al. (2025)
2020 Subcutaneous injection (SC)	First absorbed through subcutaneous tissue, then enters systemic circulation	Short	More suitable for immune-related diseases, especially wound healing	Moderate	Su et al. (2025), Fröhlich (2021), Esmaeili et al. (2022), Ai et al. (2024)

Intravenous (IV) Injection: The most widely used route, offering rapid systemic circulation. However, EVs exhibit a relatively short plasma half-life due to hepatic and renal clearance (Liu et al., 2017). However, *in vivo* neuroimaging has demonstrated that, compared with intravenous injection, EVs can cross the blood-brain barrier (BBB) more effectively after intranasal administration (Guy and Offen, 2020). Near-infrared imaging further supports this, as the imaging results show that DiR-labeled EVs can be delivered to the brain after intranasal administration, while tail vein injection mainly leads to their accumulation in the liver and kidneys (Liang et al., 2020). However, Hong et al. showed through *in vivo* imaging after intravenous administration that EVs can effectively cross the BBB and have a higher fluorescence density in the mouse brain (Hong et al., 2023). Preclinical data indicate that in acute respiratory distress syndrome, the delivery of EVs via the intravenous and intratracheal routes has similar efficacy (Zhu et al., 2014). The study by Fröhlich et al. shows that MSC-EVs are a promising option for both intravenous injection and inhalation therapy. Because they can cross the epithelial barrier better, have stronger stability, and have less procoagulant effect, but the advantages of the inhalation administration route are greatly reduced (Fröhlich, 2021). After subcutaneous injection of EVs, they will first diffuse in the local tissues and interact with surrounding cells. Then, some of the EVs can enter the circulatory system through local capillaries or lymphatic vessels, accumulate in the connected lymph nodes, and then be distributed to other parts (Sakurai et al., 2023). Therefore, subcutaneous injection of EVs can be used for research in immune-related disease models. Yang et al. subcutaneously injected EVs into the skin wounds of mice, which can effectively promote the healing of skin injuries (Yang J. et al., 2024). Currently, most EVs can be used for local administration, but the use of subcutaneous injection as an administration route has not been approved (Tawanwongsri and Vachiramon, 2024).

In recent years, significant progress has been made in biomaterials. Drug delivery research shows that hydrogels have attracted interest due to their excellent biocompatibility, degradability, and processability. In the study of various diseases, the encapsulation of EVs in hydrogels may help regulate the activity of EVs *in vivo*, thereby enhancing the therapeutic effect of EVs. Hydrogel-encapsulated EVs provide an alternative to traditional intravenous, arterial, or intramuscular injection methods (Farahzadi et al., 2024). For example, EVs are encapsulated in gelatin methacryloyl that covers the surface of the heart, and local administration may greatly increase the retention rate of EVs (Tang et al., 2022).

In conclusion, due to the many differences between studies, it is difficult to determine the advantages of the administration routes of EVs. These differences include variations in the production and preparation of EVs, uncertainties in issues related to administration, and differences between disease stages.

### 4.2 Biodistribution

EVs exhibit tissue-specific tropism *in vivo*, influenced by their cellular origin and surface modifications (Wang Y. et al., 2023). Advanced labeling techniques enable precise tracking of EVs biodistribution and spatial-temporal dynamics.

#### 4.2.1 Lipophilic fluorescent dye labeling

Carbonaceous dyes are a class of lipophilic fluorescent dyes that can be used for staining cell membranes and other lipid-soluble biological structures. When they bind to the lipid membrane, the fluorescence intensity is greatly enhanced, and this type of dye has a high quenching constant and a long excited state lifetime. Once the lipid bilayer is stained, these dyes will diffuse across the entire membrane, and at the optimal concentration, they can be applied to the entire membrane structure of EVs. Common carbocyanine dyes include DiIC_18_(5) (DiD), DiIC_18_(3) (DiI), DiOC_18_(3) (DiO), and DiIC_18_(7) (DiR) (Bao et al., 2023). After Zhang et al. labeled EVs with DiR dye and injected them into mice via the tail vein, they could observe and measure the distribution of EVs in the organs of the mice, most EVs are first distributed to the lungs and liver through the circulatory system, and can achieve retention in lung tissues (Zhang et al., 2024). DIO staining can be used to examine the uptake of EVs by human lung microvascular endothelial cells (Sun et al., 2023). PKH67 and PKH26 are also lipophilic fluorescent dyes. PKH67 and PKH26 tend to form more aggregated micelles than DiI, but an excessive amount of PKH26 may damage the structure of EVs (Chen et al., 2023). Jiang et al. isolated EVs from the representative epidermal cells of psoriasis-like mice, labeled them with the green fluorescent dye PKH67, and injected them into the back of psoriasis-like mice through microneedles to observe the effect of EVs on the pathogenesis of psoriasis (It localizes in the epidermis approximately 48 h after *in situ* injection) (Jiang S. et al., 2024).

#### 4.2.2 Radioisotope labeling

Radioisotope-labeled compounds can be used for *in vivo* tracing of EVs by introducing radioisotopes into EVs and utilizing nuclear medicine imaging techniques (such as positron emission tomography; single-photon emission computed tomography). This method has high sensitivity and specificity, but it requires professional nuclear medicine equipment and protective measures (Jiang W. et al., 2024). The main advantages of this method include the wide availability of radioisotopes and the preservation of the morphology of EVs after labeling (Almeida et al., 2020). Chung directly labeled EVs with the radioisotope Tc-99m and evaluated the *in vivo* tracking of EVs through single-photon emission computed tomography. The EVs were mainly absorbed by the liver and spleen (Chung et al., 2024). Lazaro-Ibáñez et al. covalently linked diethylenetriaminepentaacetic dianhydride to the surface of EVs and labeled EVs with indium-111 (111In3+). This labeling method showed a high radioactive labeling efficiency for EVs and enabled the observation of the specific situation in mice. EVs rapidly accumulate in the peri-abdominal area including the liver, spleen, and kidneys, and the accumulation in these organs is retained at the subsequent time points of 4 h and 24 h (Lázaro-Ibáñez et al., 2021).

#### 4.2.3 Bioluminescence imaging (BLI)

BLI is a technique used to visualize the physiological change processes in animals. Based on the bioluminescence phenomenon, researchers discovered the mechanism by which luciferase reacts with luciferin to generate light and developed BLI. BLI allows for multiple imaging sessions without euthanizing the animals to assess the changes in physiological processes over time. After injecting the luciferin substrate, the luciferase in the body interacts with the luciferin to produce light of a specific wavelength, enabling observation (Li S. et al., 2023). Villa et al. used BLI to confirm the targeting and delivery capabilities of EVs, in the absence of tumors, canine glioma-derived vesicles are capable of crossing the BBB in healthy mice, with a propensity for accumulation within the brain tissue (Villa et al., 2024). Bioluminescent reporter genes are derived from light-producing organisms, including Gaussia luciferase (GLuc) or Renilla reniformis (RLuc), etc. They have been used for the tracking of EVs. BLI has a wide range of applications and high sensitivity (Liu et al., 2022).

#### 4.2.4 Magnetic resonance imaging (MRI)

MRI is mainly a medical imaging technique, suitable for the non-invasive visualization of the body’s anatomy and physiology in diseases and health conditions. An MRI scanner uses magnetic fields, electric fields, and radio waves to generate images of organs and body structures. By using different MRI pulse sequences, MRI can provide detailed anatomical images that reflect the characteristics of tissues (Hussain et al., 2022). In addition to its use in detailed tissue imaging, MRI can also be applied to track and monitor EVs *in vivo*, thereby enhancing their visibility in biological systems. However, MRI is not the best choice for imaging the heart and gastrointestinal tract (Arifin et al., 2022).

EVs have been labeled with paramagnetic gadolinium (Gd)-based contrast agents. Gd has been bound to phospholipids and integrated into EVs derived from macrophages or MSCs (Rayamajhi et al., 2020; Abello et al., 2019). The process of inserting the Gd contrast agent into the EVs membrane occurs during extrusion, and there will be slight changes in its surface charge, size distribution, and morphology. Ultrasmall superparamagnetic iron oxide nanoparticles (USPIOs) are currently the most widely used negative contrast agents for labeling EVs and can be used for histological verification *in vitro* or *ex vivo*. In rodent models, EVs derived from MSCs, macrophages, or other stem cells labeled with USPIOs can be detected *in vivo* and exhibit signals, MRI revealed that intravenously administered magneto-EVs possess the homing function to injury sites, including the kidneys and heart (Han et al., 2021).

#### 4.2.5 Computed tomography (CT)

The imaging principle of CT is to use an X-ray beam to perform continuous cross-sectional scans around a certain part of the human body. It has the characteristics of fast scanning speed and clear images, and can be used for the examination of various diseases (Albers and Kinnaird, 2024). Gold nanoparticles have become the most widely used CT probes due to their excellent X-ray absorption and biological inertia. Researchers used gold nanoparticles to label EVs and conducted longitudinal and quantitative tracking of EVs in A431 tumor-bearing mice through CT, demonstrating that MSC-EVs derived from the umbilical cord may have superior tumor-targeting therapeutic capabilities (Cohen et al., 2021). The CT imaging technique was used to obtain the biodistribution curves of EVs in the major organs of adult rhesus monkeys, thereby describing the biodistribution and *in vivo* kinetics of EVs. They are able to cross the biological barriers and appear in larger quantities in the brain (Haney et al., 2022). CT imaging has been used to study the effect of EVs on tumor targeting, but CT examination has a certain level of radiation (Huang P. et al., 2024).

### 4.3 Clearance mechanisms

The clearance of EVs is a finely regulated process critical for maintaining intercellular communication, disease progression, and physiological homeostasis.

#### 4.3.1 Macrophage mediated phagocytosis

As an important part of the body’s immune system, macrophages have a powerful phagocytic function. They can recognize and phagocytize EVs (Yang et al., 2022), and then, through digestive and degradation mechanisms such as lysosomes, they bind to lysosomes and decompose and remove EVs (Buratta et al., 2020). Methyl-β-cyclodextrin may promote the decomposition of the vesicle contents, thus leading to a reduction in EVs. Previous studies have shown that in the AD model, Aβ-related EVs are sorted into lysosomes and subsequently degraded in microglia (Pantazopoulou et al., 2023).

#### 4.3.2 Renal filtration

Small EVs undergo glomerular filtration due to their size and negative surface charge, while larger EVs remain in systemic circulation. Charge-selective glomerular pores preferentially clear anionic EVs, whereas neutral or cationic EVs exhibit prolonged circulation times (Alli, 2023).

### 4.4 Safety profile

EVs retain the bioactive properties of parental cells while circumventing risks associated with cell-based therapies, such as tumorigenicity and vascular occlusion, owing to their non-replicative nature (Xia et al., 2024). A prospective, single-arm, open-label Phase I trial conducted at the State Key Laboratory of Ophthalmology (Guangzhou, China) demonstrated the safety and efficacy of umbilical cord-derived MSC-EVs in treating refractory graft-versus-host disease (GvHD)-associated dry eye disease (DED). Topical administration (4× daily for 14 days) significantly reduced ocular surface inflammation, accelerated corneal epithelial regeneration, and showed no systemic adverse effects (Zhou et al., 2022). In addition, MSC-EVs cannot self-replicate, which avoids many of the risks associated with stem cell therapy. At the same time, they exhibit beneficial effects such as activating the immune system and prolonging the therapeutic effect (Yin et al., 2023).

By understanding the processes such as the distribution and metabolism of EVs *in vivo*, it is necessary to determine the appropriate dosage, administration frequency, and administration route to improve the efficacy of EV-based drugs and reduce adverse reactions. There are still many aspects that need in-depth exploration in the study of the pharmacokinetics of EVs. With the continuous advancement of technology, a clearer and more accurate understanding of their dynamic changes *in vivo* will be achieved, thereby better promoting the clinical applications related to EVs.

## 5 Clinical investigations of MSC-EVs

A growing body of evidence shows that MSC-EVs have cell therapeutic bioactivities similar to their parental cells, while avoiding the safety risks of live stem cell administration (Yang R. et al., 2024). ClinicalTrials.gov data reveals a rising number of registered clinical trials using MSC-EVs as interventions. As of 25 April 2025, a search for “MSC-exosomes” and “MSC-EVs” on the platform identified 28 clinical studies in total (Table 5).

**TABLE 5:** Clinical trials of MSC-derived EVs (MSC-EVs).

Order	NCT Number	Study title	Study status	Conditions	Phases	First Posted	Source	Locations	Study results
1	NCT04356300	Exosome of Mesenchymal Stem Cells for Multiple Organ Dysfuntion Syndrome After Surgical Repaire of Acute Type A Aortic Dissection	NOT_YET_RECRUITING	Multiple Organ Failure	NA	2020/4/22	Umbilical cord	China	NO
2	NCT05871463	Effect of Mesenchymal Stem Cells-derived Exosomes in Decompensated Liver Cirrhosis	RECRUITING	Decompensated Liver Cirrhosis	PHASE2	2023/5/23	Umbilical cord	Tehran	NO
3	NCT05261360	Clinical Efficacy of Exosome in Degenerative Meniscal Injury	RECRUITING	Knee; Injury, Meniscus (Lateral) (Medial)|Meniscus Tear|Meniscus Lesion|Meniscus; Degeneration|Meniscus; Laceration|Meniscus Injury, Tibial|Knee Injuries|Knee Pain Swelling|Arthralgia	PHASE2	2022/3/2	Autologous Synovial Fluid	Turkey	NO
4	NCT05808400	Safety and Efficacy of Umbilical Cord Mesenchymal Stem Cell Exosomes in Treating Chronic Cough After COVID-19	RECRUITING	Long COVID-19 Syndrome	EARLY_PHASE1	2023/4/11	Umbilical cord	China	NO
5	NCT05216562	Efficacy and Safety of EXOSOME-MSC Therapy to Reduce Hyper-inflammation In Moderate COVID-19 Patients	UNKNOWN	SARS-CoV2 Infection	PHASE2|PHASE3	2022/1/31	Not mentioned	Indonesia	NO
6	NCT03437759	MSC-Exos Promote Healing of MHs	UNKNOWN	Macular Holes	EARLY_PHASE1	2018/2/19	Umbilical cord	China	NO
7	NCT05523011	Safety and Tolerability Study of MSC Exosome Ointment	COMPLETED	Psoriasis	PHASE1	2022/8/31	Not mentioned	Singapore	The results of this study were to provide the first clinical information on the drug’s safety and inform the selection of administration of exosome ointment to be evaluated in subsequent clinical studies
8	NCT05499156	Safety of Injection of Placental Mesenchymal Stem Cell Derived Exosomes for Treatment of Resistant Perianal Fistula in Crohn’s Patients	UNKNOWN	Perianal Fistula in Patients With Crohn’s Disease	PHASE1|PHASE2	2022/8/12	Placenta	Tehran	NO
9	NCT05738629	Safety and Efficacy of Pluripotent Stem Cell-derived Mesenchymal Stem Cell Exosome (PSC-MSC-Exo) Eye Drops Treatment for Dry Eye Diseases Post Refractive Surgery and Associated With Blepharospasm	NOT_YET_RECRUITING	Dry Eye Disease	PHASE1|PHASE2	2023/2/22	Pluripotent stem cells	China	NO
10	NCT05669144	Co-transplantation of Mesenchymal Stem Cell Derived Exosomes and Autologous Mitochondria for Patients Candidate for CABG Surgery	RECRUITING	Myocardial Infarction|Myocardial Ischemia|Myocardial Stunning	PHASE1|PHASE2	2022/12/30	Umbilical cord	Tehran	NO
11	NCT02138331	Effect of Microvesicles and Exosomes Therapy on β-cell Mass in Type I Diabetes Mellitus (T1DM)	UNKNOWN	Diabetes Mellitus Type 1	PHASE2|PHASE3	2014/5/14	Umbilical cord blood	Egypt	NO
12	NCT06599346	Effects of Mesenchymal Stem Cell Supernatant on Prevention and Treatment of Skin/Mucosal Injury in Hematology Patients	RECRUITING	Mucositis|Hematopoietic Stem Cell Transplantation|Chemotherapy-Induced Mucositis|Radiation-Induced Mucositis	NA	2024/9/19	Not mentioned	China	NO
13	NCT05060107	Intra-articular Injection of MSC-derived Exosomes in Knee Osteoarthritis (ExoOA-1)	UNKNOWN	Osteoarthritis, Knee	PHASE1	2021/9/28	Not mentioned	Chile	NO
14	NCT06431152	Intra-articular Injection of UC-MSC Exosome in Knee Osteoarthritis	RECRUITING	Osteo Arthritis Knee	EARLY_PHASE1	2024/5/28	Umbilical cord	Chile	NO
15	NCT03384433	Allogenic Mesenchymal Stem Cell Derived Exosome in Patients With Acute Ischemic Stroke	UNKNOWN	Cerebrovascular Disorders	PHASE1|PHASE2	2017/12/27	Not mentioned	Tehran	NO
16	NCT06598202	Exploring Nasal Drop Therapy With Small Extracellular Vesicles for ALS	RECRUITING	Amyotrophic Lateral Sclerosis	PHASE1|PHASE2	2024/9/19	Umbilical cord	China	NO
17	NCT04798716	The Use of Exosomes for the Treatment of Acute Respiratory Distress Syndrome or Novel Coronavirus Pneumonia Caused by COVID-19	NOT_YET_RECRUITING	COVID-19|Novel Coronavirus Pneumonia|Acute Respiratory Distress Syndrome	PHASE1|PHASE2	2021/3/15	Not mentioned	American	NO
18	NCT04173650	MSC EVs in Dystrophic Epidermolysis Bullosa	NOT_YET_RECRUITING	Dystrophic Epidermolysis Bullosa	PHASE1|PHASE2	2019/11/22	Not mentioned	American	NO
19	NCT04850469	Study of MSC-Exo on the Therapy for Intensively Ill Children	WITHDRAWN	Sepsis|Critical Illness		2021/4/20	Not mentioned	China	NO (Revoke)
20	NCT05402748	Safety and Efficacy of Injection of Human Placenta Mesenchymal Stem Cells Derived Exosomes for Treatment of Complex Anal Fistula	UNKNOWN	Fistula Perianal	PHASE1|PHASE2	2022/6/2	Placenta	Tehran	NO
21	NCT03608631	IExosomes in Treating Participants with Metastatic Pancreas Cancer with KrasG12D Mutation	ACTIVE_NOT_RECRUITING	KRAS NP_004976.2:p.G12D|Metastatic Pancreatic Adenocarcinoma|Pancreatic Ductal Adenocarcinoma|Stage IV Pancreatic Cancer AJCC V8	PHASE1	2018/8/1	Not mentioned	American	NO
22	NCT04602442	Safety and Efficiency of Method of Exosome Inhalation in COVID-19 Associated Pneumonia	UNKNOWN	COVID-19|SARS-CoV-2 PNEUMONIA|COVID-19	PHASE2	2020/10/26	Not mentioned	Russia	NO
23	NCT04491240	Evaluation of Safety and Efficiency of Method of Exosome Inhalation in SARS-CoV-2 Associated Pneumonia	COMPLETED	COVID-19|SARS-CoV-2 PNEUMONIA|COVID-19	PHASE1|PHASE2	2020/7/29	Not mentioned	Russia	It is assumed that the inhalation method of injection of exosomes will speed up the rehabilitation of patients, reduce the volume of pulmonary tissue lesions and reduce the time of stay of patients in hospital conditions
24	NCT05243368	Evaluation of Personalized Nutritional Intervention on Wound Healing of Cutaneous Ulcers in Diabetics	RECRUITING	Foot, Diabetic	NA	2022/2/17	Not mentioned	Spain	NO
25	NCT04388982	the Safety and the Efficacy Evaluation of Allogenic Adipose MSC-Exos in Patients With Alzheimer’s Disease	UNKNOWN	Alzheimer Disease	PHASE1|PHASE2	2020/5/15	Fat	China	NO
26	NCT06812637	Efficacy and Safety of Wharton’s Jelly-Derived Mesenchymal Stem Cell Exosomes in the Treatment of Diabetic Foot Ulcers: a Double-blinded Randomized Controlled Clinical Trial	COMPLETED	Diabetic Foot Ulcer (DFU)|Exosomes	PHASE1	2025/2/6	Warton Jelly	Egypt	The outcomes of this research could not only enhance healing rates but also significantly improve the quality of life for individuals suffering from chronic diabetic foot ulcers
27	NCT06919380	Nebulized MSC-Exos for Anti-MDA5+ RP-ILD: Safety and Efficacy Trial	RECRUITING	Anti-MDA5 Positive Dermatomyositis-Associated RP-ILD|Rapidly Progressive Interstitial Lung Disease	PHASE1	2025/4/9	Not mentioned	China	NO
28	NCT06896747	Evaluating Mechanically Engineered Stem Cell Exosomes for Treating Endometrial Injury: a Clinical Study	RECRUITING	Thin Endometrial Lining|Female Infertility|Intrauterine Adhesions	PHASE1|PHASE2	2025/3/26	Umbilical cord	China	NO

These studies cover various conditions: 5 focus on pneumonia, 3 on diabetes-related diseases, and 2 on knee osteoarthritis. Single studies address AD, meniscus injury, multiple organ failure, decompensated liver cirrhosis, macular hole, psoriasis, perianal fistula (with two separate studies), dry eye syndrome, myocardial infarction, mucositis, cerebrovascular diseases, ALS, dystrophic epidermolysis bullosa, sepsis, metastatic pancreatic adenocarcinoma, lung diseases, and endometrial injury.

Notably, neurological diseases such as AD, ALS, and cerebrovascular diseases are included. These trials not only highlight the potential of MSC-EVs in treating neurological diseases but also provide preliminary evidence for their safety in clinical settings.

## 6 Therapeutic applications of MSC-EVs in neurological disorders

Neurological diseases are a group of diseases characterized by the breakdown of the structure and function of neural networks, as well as impaired memory, cognitive, behavioral, sensory, and motor functions. They include various conditions such as AD, PD, Multiple sclerosis, etc. Neurological diseases are the leading cause of disability and the second leading cause of death, and they also impose a heavy economic burden (Cheng et al., 2024). Globally, approximately 30% of people may experience neurological problems at some point in their lives (Salehpour et al., 2024). Stroke, migraine, AD, and other dementias are the most common neurological causes of disability. In the past few years, the number of deaths has climbed by 30%, and the number of years of life lost due to disability has decreased by 15% (Feigin et al., 2020). MSC-EVs mimic certain properties of MSCs, including their ability to suppress the immune system and promote tissue repair. MSC-EVs carry a large number of biomolecules, such as proteins (extracellular, intracellular, enzymes, receptors), lipids, and nucleic acids (Hamidi et al., 2024). MSC-EVs exhibit low immunogenicity, high heterogeneity, and the ability to promote intercellular signaling and regulate the extracellular matrix, significantly enhancing their involvement in various physiological and pathological processes. Compared with MSCs, MSC-EVs offer several advantages for clinical applications (Karnas et al., 2023). MSC-EVs have a wide range of therapeutic applications in the treatment of neurological diseases. MSC-EVs have been found to be beneficial for neurological diseases caused by AD, PD, Amyotrophic Lateral Sclerosis (ALS), Peripheral Nerve Injury (PNI), Spinal Cord Injury (SCI), and hearing loss.

### 6.1 Umbilical cord derived MSC-EVs (UCMSC-EVs)

AD is a progressive neurodegenerative disease characterized by mitochondrial dysfunction, the accumulation of β-amyloid plaques, and hyperphosphorylated tau tangles in the brain, leading to memory loss and cognitive deficits. EVs contain proteins, lipids, microRNAs, and other molecules that can affect the functions of neighboring cells and promote regeneration (Belousova et al., 2024). EVs derived from UCMSCs have been well demonstrated to cross the BBB and target their active components (such as proteins, miRNAs, etc.) to the damaged areas of the central nervous system, becoming a research hotspot in the field of neural therapy (Hu et al., 2024).

UCMSC-EVs can improve the occurrence of Aβ accumulation and neuroinflammation in AD animals by regulating the activity of microglia (Fallahi et al., 2024). PD is a common neurodegenerative disease that has received extensive attention. However, the current clinical treatments can only alleviate its symptoms and cannot effectively protect dopaminergic neurons. UCMSC-EVs enhance the cellular antioxidant defense mechanism by activating the nuclear factor erythroid 2-related factor 2 signaling pathway, protecting neurons from damage (Wang Z. et al., 2024). Compared with EVs derived from bone marrow mesenchymal stem cells (BMSC-EVs), UCMSC-EVs are richer in the regulatory potential related to the cell cycle, DNA replication, and repair, which can improve the motor ability of PD model mice and enhance the recovery of the olfactory and motor functions of the mice (Huang W. et al., 2024). Depression is characterized by neuroinflammation and neurodegeneration. UCMSC-EVs exhibit antidepressant effects by inhibiting the neuroinflammation of M1 microglia (Li S. et al, 2024). Ischemic stroke is one of the cardiovascular diseases globally, characterized by a high incidence and mortality rate. After a period of long-term ischemia caused by a stroke, the restoration of blood flow will trigger inflammation and the excessive production of reactive oxygen species (ROS), leading to neuronal cell death and brain damage. This phenomenon is called cerebral ischemia/reperfusion injury (CIRI) (Datta et al., 2020). UCMSC-EVs have certain therapeutic potential in alleviating CIRI in various cell types, including neuronal cells (Wang C. C. et al., 2024). TBI is a major cause of neurological dysfunction, and the current treatment methods have limited effects. UCMSC-EVs can improve the damaged microenvironment by inhibiting the injury-induced excessive activation of microglia and the neuroinflammatory response, effectively alleviating TBI (Cui et al., 2024). The results of a first-in-human, single-arm, open-label, phase I clinical trial indicate that the intrathecal administration of UCMSC-EVs is safe for patients with subacute SCI, and there are significant improvements in the subscales of respiratory and sphincter management (Akhlaghpasand et al., 2024). Multiple sclerosis is an immune-mediated central nervous system disease characterized by causing inflammation, demyelination, and neuronal degeneration. Common symptoms include motor and sensory disorders, fatigue, pain, visual impairment, and cognitive dysfunction (Kråkenes et al., 2024). UCMSC-EVs can regulate the immune response of mice with experimental autoimmune encephalomyelitis (an Multiple sclerosis model) by promoting the expression of Lag-3 on CD4+/Foxp3 Tregs and reducing the infiltration of immune cells in the hypothalamus (Mohammadzadeh et al., 2024). Wharton’s jelly (WJ) is a major source of MSCs from the umbilical cord. WJ-MSCs are isolated from umbilical cord tissue through a non-invasive and painless procedure, and their EVs are also referred to as UCMSC-EVs (Patel et al., 2024; Drobiova et al., 2023). WJ-MSC-EVs can protect hippocampal neurons from oxidative stress and damage induced by Aβ oligomers, an effect associated with the transfer of enzymatically active catalase contained in these EVs (Bodart-Santos et al., 2019). After intranasal administration of WJ-MSC-EVs to mice, Zhdanova et al. found that the spatial memory of olfactory bulbectomized mice was improved, with labeled EVs detected in the hippocampus and cortex (Zhdanova et al., 2021).

### 6.2 Bone marrow derived MSC-EVs (BMSC-EVs)

SCI severely impairs the quality of life of patients, manifesting as complications such as motor dysfunction, neuropathic pain, disorders of urination and defecation, and sexual dysfunction (Yang S. et al., 2024). BMSC-EVs show promising potential for the repair of SCI, a neurological disease. The research results of Xu indicate that BMSC-EVs have improved SCI in rats, manifested as the recovery of motor function, the alleviation of pathological conditions, and the reduction of apoptosis, inflammatory response, and oxidative stress. BMSC-EVs promote SCI repair through miR-497-5p/TXNIP/NLRP3, which may be a target for alleviating SCI-related nerve damage (Xu et al., 2024). Ischemic stroke, caused by a clot disrupting the blood supply to the brain, is an important cause of long-term neurological disability and death among adults worldwide (Fan et al., 2023). When intranasal BMSC-EVs treatment is combined with drug treatment, adult rats with transient middle cerebral artery occlusion stroke exhibit significant functional recovery in the neurological severity score and the beam balance test, with the striatum and cortex being greatly affected (Gomez-Galvez et al., 2024). ALS is a fatal neurodegenerative disease of motor neurons, and it is estimated that 6.6 out of every 100,000 people in the United States suffer from this disease. The study by Crose et al. shows that BMSC-EVs treatment has no serious side effects, has a certain degree of safety, and may have the potential to delay the progression of ALS (Crose et al., 2024). Sensorineural Hearing Loss (SNHL) is one of the most prevalent sensory deficits, having a severe negative impact on patients. SNHL usually involves damage to the central auditory pathway. Intratympanic injection of BMSC-EVs is a specific and effective strategy for the treatment of neurological hearing loss and can minimize surgical trauma and systemic side effects (Chen et al., 2024).

### 6.3 Adipose derived MSC-EVs (ADSC-EVs)

As outlined by the World Health Organization (WHO), Glioblastoma Multiforme (GBM) is a grade 4 central nervous system tumor and a fatal cancer with a high incidence and mortality rate. EVs derived from ADSCs contain microRNA-4731-5p (miR-4731-5p), and its expression has good antitumor properties against GBM (Babaei et al., 2024). Intranasal administration of ADSCs-EVs can improve the motor performance of ALS mice and inhibit muscle atrophy (Turano et al., 2024). ADSCs, especially those from hypoxic-preconditioned ADSCs-MSCs, possess therapeutic properties that can promote spinal cord repair in SCI (Shao et al., 2024). Spinal Muscular Atrophy (SMA) is an autosomal recessive neuromuscular disease. Its typical symptoms include hypomyotonia, muscle atrophy, and weakness caused by denervation, until the muscles of the upper and lower limbs as well as the trunk muscles are eventually paralyzed. Relevant studies have shown that when treating SMNΔ7 mice (a model of SMA type II), ADSCs-EVs may play a neuroprotective role at the central level and in terms of motor function (Virla et al., 2024). Glaucoma is a progressive neurodegenerative disease and one of the most common causes of irreversible blindness. Increasing evidence indicates that neuroinflammation is associated with the pathological process of glaucoma (Ji et al., 2024). Some researchers believe that compared with MSC-EVs from other sources, ADSCs-EVs have a better effect in immune inflammation regulation (Tham et al., 2014). Ji et al. believe that ADSCs-EVs can reduce the expression of microglia-related CD68, CCL2, and TLR4 in the retina and optic nerve of mice, and they are potential therapeutic candidates or adjuvant treatment options for glaucoma (Ji et al., 2024).

### 6.4 Placental derived MSC-EVs (PMSC-EVs)

EVs derived from PMSCs contain proteins with proliferative-promoting properties (FGF-2, PDGF, and HGF), anti-catabolic properties (TIMP-1 and TIMP-2), and anti-inflammatory properties (sICAM-1) (Shipman et al., 2024). Intrathecal injection of PMSC-EVs during the acute phase of SCI in female rats can improve functional recovery, reduce injury-related pathological changes, and prevent chronic diseases after SCI. Therefore, PMSC-EVs have certain neuroprotective and anti-apoptotic potentials (Soleimani et al., 2024). Intravenous injection of PMSC-EVs can promote the activation of endogenous neural progenitor cells and the ability of neurogenesis, and facilitate the recovery of motor and autonomic nerve functions damaged by SCI (Zhou et al., 2021). Duan et al. found that *in-situ* injection of PMSC-EVs can inhibit the occurrence of inflammation and reduce oxidative stress (Duan et al., 2020). High-dose PMSC-EVs can improve motor function in EAE, a mouse model of Multiple sclerosis, and this may be linked to neuroprotective mechanisms (Clark et al., 2019). The placenta, like the umbilical cord, comes from mesenchymal tissues in embryonic development. But because of its complex structure, isolating and purifying PMSCs from it needs more refined procedures (Wang et al., 2025). There is less research on PMSC-EVs compared with UCMSC-EVs. Going forward, it is important to dig deeper into their neuroprotective mechanisms and clinical application value.

### 6.5 Dental pulp derived MSC-EVs (DPSC-EVs)

Subarachnoid Hemorrhage (SAH) caused by an aneurysm is a severe subtype of stroke, accounting for approximately 5% of all stroke cases. Compared with other types of strokes, SAH is characterized by a younger onset age, higher morbidity and mortality rates (Liang et al., 2024). EVs derived from DPSCs can be extracted from human wisdom teeth or deciduous teeth. The extraction method is minimally invasive, safe, and free of ethical issues, and they exhibit immunomodulatory properties (Vu et al., 2022). DPSCs-EVs can inhibit the activation of microglia and the secretion of pro-inflammatory cytokines after SAH, and significantly alleviate the overall brain edema and nerve damage in rats (Liang et al., 2024). PNI is a common and destructive neurodegenerative disease, which easily leads to motor and sensory dysfunctions in patients. DPSCs-EVs can promote axonal regeneration and remyelination after PNI by increasing the proliferation, migration, and secretion of neurotrophic factors (Chai et al., 2024). Recent studies show DPSC-EVs promote axonal regeneration and functional recovery in injured mouse sciatic nerves by enhancing Schwann cell proliferation/migration and upregulating c-JUN, Notch1, GFAP, and SRY-box 2 (Mao et al., 2019). In ischemic stroke-related CIRI, DPSC-EVs reduce cerebral edema, infarct volume, and neurological deficits by alleviating neuronal apoptosis—likely via miR-877-3p interacting with Bclaf1 (Miao et al., 2024). A single systemic administration also lessens infarct size, neuroinflammation, and impairment (Ji et al., 2019). DPSC-EVs, rich in VEGF and MCP-1, mitigate Aβ cytotoxicity, boost cell viability, and regulate Bcl-2/Bax to inhibit apoptosis (Ahmed et al., 2016). Their parent DPSCs’ high proliferation and neurogenic capacity support potential in craniofacial/neural regeneration (Ahmad et al., 2025). However, DPSC extraction (requiring tooth removal) is impractical and unethical except for wisdom/orthodontic teeth.

**FIGURE 2:**
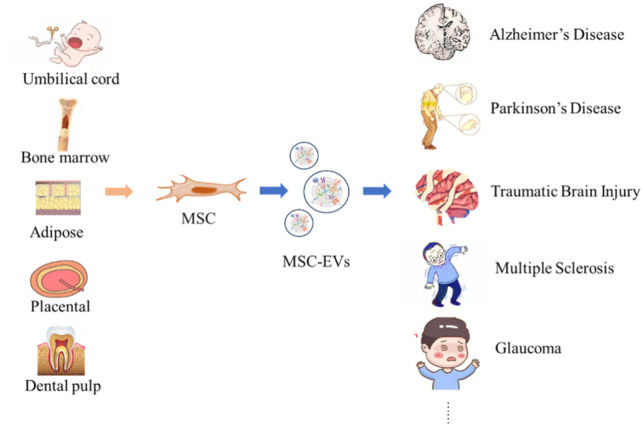
Neurological Disorders Targeted by MSC-EVs from Diverse Origins. MSC can be obtained from tissues such as umbilical cord, bone marrow, adipose tissue, placenta, and dental pulp. MSC secrete MSC-EVs, and these vesicles show potential in the therapeutic application for neurological diseases such as AD, PD, TBI, Multiple sclerosis, and GLA.

This article summarizes MSC-EVs from five different sources (Table 6), which provide abundant resources and broad application prospects for the treatment and research of numerous neurological diseases (Figure 2) (Li Y. et al., 2024; Rather et al., 2023; Santilli et al., 2024; Mushahary et al., 2018).

**TABLE 6:** Applications of different MSC-EVs in neurological disorders.

Source	Advantages	Therapeutic mechanism	Surface markers
Umbilical Cord/ Wharton’s jelly	Obtained in a non-invasive manner, free from ethical and legal restrictions, and capable of synthesizing and secreting various trophic factors and cytokines	Nrf2/ CD4^+^/Foxp3 Tregs/ miR-1228-5p/ NF-κB/ p38 MAPK	Positive: CK8, CK18, CK19, CD10, CD13, CD29, CD44, CD73, CD90, CD105, CD106, HLA-I, HLA-II; Negative: CD14, CD31, CD33, CD34, CD45, CD38, CD79, CD133, vWF, HLA-DR
Bone Marrow	Can directly participate in tissue repair by differentiating into various cells	NLRP3/ miR-497-5p/ TXNIP/ NLRP3	Positive: SH2, SH3, CD29, CD44, CD49e, CD71, CD73, CD90, CD105, CD106, CD166, CD120a, CD124; Negative: CD34, CD45, CD19, CD3, CD31, CD11b, HLA-DR
Adipose	Abundant in adipose tissue, with easy accessibility, high proliferative potential, and strong self-renewal ability	miR-4731-5p/ miR-138-5p/ GPX4/ TLR4/ MAPK/ NF-κB	Positive: CD13, CD29, CD44, CD73, CD90, CD105, CD166, HLA-I, HLA-ABC; Negative: CD10, CD14, CD24, CD31, CD34, CD36, CD38, CD45, CD49d, CD117, CD133, SSEA4, CD106, HLA-II, HLA-DR
Placenta	Derived from human amniotic membrane, originating from the ectoderm during embryonic development, with low immunogenicity and anti-inflammatory properties	MEK/ERK/CREB	Positive: CD29, CD44, CD73, CD90, CD105; Negative: CD45, CD34, HLA-DR
Dental Pulp	Derived from neural crest, consisting of cells derived from ectoderm (which is also the origin of mature neurons)	miRNA-197-3p/ FOXO3/ miR-122-5p/ P53	Positive: CD29, CD44, CD90, CD105, SH2, SH3, HLA-DR, CD117, CD146, DPSC-EZ, DPSC-OG; Negative: CD10, CD14, CD34, CD45, HLA-DR, Stro-1, NGFR

## 7 Conclusion

MSC-EVs have emerged as promising alternatives to whole-cell therapies, leveraging their unique advantages such as BBB penetrance, low immunogenicity, and high biosafety. These attributes position MSC-EVs at the forefront of biomedical and clinical research, offering novel therapeutic strategies for refractory diseases (Rashidi et al., 2022; Zanirati et al., 2024). Despite their transformative potential, the clinical translation of MSC-EVs faces significant challenges. A critical hurdle lies in the standardization and validation of isolation protocols to ensure inter-batch consistency and reproducibility (Ma et al., 2025). Current methods including ultracentrifugation, differential centrifugation, density gradient centrifugation, ultrafiltration, AEC, SEC, polymer precipitation, immunomagnetic beads, and microfluidics each present trade-offs in purity, yield, and scalability. No single technique universally addresses all research needs, necessitating integrated approaches for optimal EVs recovery and functionality.

Secondly, this article analyzes various identification techniques and pharmacokinetics of MSC-EVs. In clinical applications, although MSC-EVs do not pose the same ethical and immunogenic dilemmas as stem cells, their administration methods still need to be further standardized. Different diseases models usually require different administration methods. Before clinical application, an appropriate administration method of EVs must be selected to evaluate its therapeutic effect and potential toxic and side effects, thus promoting subsequent scientific research.

Finally, we summarize the relevant research on the therapeutic applications of MSC-EVs in various neurological diseases. Up to now, the Food and Drug Administration (FDA) of the United States has not approved any MSC-EVs-based therapies for clinical use. MSC-EVs from different parental sources are being studied in various preclinical and clinical research to understand their potential in treating a range of neurological diseases (such as AD, PD, SCI, ALS, etc.), with the hope of providing guidance for future clinical research.

In conclusion, MSC-EVs have good therapeutic potential in treating nerve injuries and neurological diseases, but several challenges must still be addressed before clinical application. In the future, continuous research is crucial for fully unleashing the therapeutic potential of MSC-EVs and overcoming the technical and regulatory barriers they face for effective use in clinical practice.
